# Does ‘Time Heal all Wounds?’ The Prevalence and Predictors of Prolonged Grief Among Drug-Death Bereaved Family Members: A Cross-Sectional Study

**DOI:** 10.1177/00302228221098584

**Published:** 2022-04-28

**Authors:** Kristine B. Titlestad, Kari Dyregrov

**Affiliations:** 1 Faculty of Health and Social Sciences, 1657 Western Norway University of Applied Sciences, Bergen, Norway

**Keywords:** bereavement, drug-related death, family, prolonged grief, cross-sectional

## Abstract

Despite rising rates of drug-related deaths (DRDs), the consequences of DRDs for bereaved family members are scarcely investigated. This study aimed to estimate the prevalence of prolonged grief (PG) symptoms in bereaved family members after DRDs, identify predictors of PG and examine whether symptom levels decrease with time. A cross-sectional design based on survey data from parents (*n* = 93), siblings (*n* = 78), children (*n* = 24) and other family members (*n* = 39) was conducted (*n* = 234). Descriptive analyses, a multivariate linear regression, and ANOVA were performed. 60 family members (26%) suffered from high levels of PG symptoms after DRDs (parents 31.2%, siblings 21.8%, children 20.9%). The strongest associations were found between a high level of symptoms and ‘months since the loss’, ‘suicidal thoughts’ and ‘withdrawal from others’. The ANOVA analyses showed that time does not always ‘heal all wounds’, and the bereaved who lost one to 2 years ago had the highest level of PG symptoms.

## Introduction

### The Number of Drug-Related Deaths is Increasing

Mortality associated directly or indirectly with illicit drug use is a major contributor to the number of adult deaths worldwide (European Monitoring Centre for Drugs and Drug Addiction, 2021). In the United States, drug-related deaths (DRDs) reached epidemic proportions in 2017, when the age-adjusted rate of drug overdose deaths (21.7 per 100,000) was 9.6% higher than the 2016 rate (19.8 per 100,000) (Centers for Disease Control and Prevention, 2019). Updated numbers are alarming; new figures published in the US in December 2020 showed that the number of overdose deaths increased by 18.2% from June 2019 to May 2020. The largest increase recorded from March to May 2020 coincided with measures implemented as a result of the COVID-19 pandemic (Centers for Disease Control and Prevention, 2020). This negative trend has also been observed in most European countries. More than 200,000 people die from illicit drug overdoses every year and this figure is higher when DRDs are included, e.g., deaths related to HIV, hepatitis C and infections. In Norway, with a population of five million people, there are approximately 300 DRDs annually. This is an incidence rate of approximately six cases per 100,000 and is comparable to other Scandinavian countries (European Monitoring Centre for Drugs and Drug Addiction, 2019). In 2020, Norway had the highest number of DRDs for 20 years (The Norwegian Institute of Public Health, 2021). A conservative estimate is that a death strongly impacts 10–15 people close to the deceased, which means that globally, two to two-and-a-half million people are bereaved by a DRD every year.

### Consequences for Bereaved by DRD

Research in substance use fields has focused on the relationship between substance use and complicated grief, but few studies have investigated people who are bereaved by use of substances (Parisi et al., 2019). Despite the huge number of affected individuals, a systematic review (SR) documents that there is scarce research about the consequences and strains of DRDs for close, bereaved people (Titlestad et al., 2019). However, a recent registry study was conducted in Norway, which showed that parents bereaved by DRD had a higher mortality by external causes (e.g., suicide and deaths due to substance use) and natural causes than both non-bereaved parents and parents bereaved by other causes of death (Christiansen et al., 2020). Parents bereaved by DRD had a particularly high external cause mortality in the first 2 years after the loss, the hazard ratio for the mothers 4.82 (CI = 3.11–7.47) and the fathers 2.50 (CI = 1.57–3.97). An enduring bond to the deceased and the inability to find a further meaning in life, increase the risk of a complicated grief process (Boelen et al., 2006). Christiansen et al. (2020) also indicated that bereavement by DRD is associated with adverse health outcomes and that these might be more severe than bereavement by other causes of death.

Drug-related death has been characterized as a ‘special death’ (Dyregrov et al., 2019; Guy & Holloway, 2007; Templeton et al., 2017; Titlestad, Mellingen, et al., 2020), referring to the extreme strain and ambivalence involved generally when living with a person who uses narcotics, which is then furthered by experiencing a traumatic DRD. For years pre-loss, many family members may live with a profound anxiety and constant fear that using narcotics will kill their loved ones. When death occurs, they may be worn out by the efforts to prevent the death and less capable of manage their grief process (Dyregrov et al., 2019). Processing an overload of stress due to constant preparedness in extended parenthood, as well as the perceived stigma and grief-related emotions and reactions, may lead to a ‘special grief’ (Dyregrov et al., 2019; Titlestad, Mellingen, et al., 2020).

Unnatural deaths are found to cause serious and long-lasting consequences for those who were close to the deceased person, including prolonged grief disorder (PGD), traumatic stress reactions, anxiety, depression, suicidal ideation, physical illness, social isolation and existential crises (Kristensen et al., 2012; Li et al., 2003; Rostila & Saarela, 2011; Stroebe et al., 2007). Unnatural deaths, such as DRDs, suicides and accidents, are characterized by their sudden, unexpected, violent and often self-inflicted or premature character. Rates of PGD are reported to be especially elevated in bereaved individuals following unnatural losses compared to other causes of death (Djelantik et al., 2020). About 10–15% of bereaved people develop PGD after expected losses (Lundorff et al., 2017), compared to 30–70% of bereaved people after sudden and violent losses (Djelantik et al., 2020). PGD is characterized by intense longing or yearning for the deceased, a preoccupation with the deceased and/or the circumstances of the death, and emotional pain with symptoms such as difficulties accepting the death, anger or bitterness, a feeling that life is meaningless and wanting to die (suicidal ideation) (Prigerson et al., 2009; Shear et al., 2011). To qualify for the diagnosis of PGD in the ICD-11symptoms must persist for 6 months after the loss and must be associated with functional impairment (Eisma & Stroebe, 2021; Stroebe et al., 2007). A recent study by Rosner et al. (2021) showed a prevalence of 4.2% of a representative sample in the general population when based on the strict criteria of the ICD-11. Only one study has investigated the prevalence of symptoms of prolonged grief (PG) among people bereaved after DRDs (Titlestad et al., 2021). High levels of PG symptoms were identified among parents and low levels of self-efficacy and withdrawal from others were strongly associated with high levels of grief symptoms. Therefore, to provide need-related help for the bereaved, there is a strong demand for more knowledge about grief, especially PG.

### Factors Increasing the Risk of High Levels of Prolonged Grief Symptoms

Previous research has shown that various factors might influence the prevalence, comorbidity and risks of PGD of the individual after experiencing a loss. The nature of the loss, i.e., violent losses such as homicide, suicide, accidents or disasters, increases the risk of developing PGD (Kristensen et al., 2012). Systematic reviews have documented that a short time since the loss (Hardison et al., 2005; He et al., 2014; Lobb et al., 2010), previous losses, traumatic exposure (Lobb et al., 2010), degree of closeness to the deceased, lack of preparation for the death and high distress at the time of the death are predictive of PG (Hardison et al., 2005; Lundorff et al., 2017). Younger age of the deceased and being a woman have also been found to be predictive of PG (Hardison et al., 2005). Using the diagnostic tool Prolonged Grief Disorder-13 (PG-13) in a population of bereaved adults in China, He et al. (2014) found that increased PG-13 scores were significantly related to the participant’s younger age, a short time since the loss and a younger age of the deceased. Being divorced/widowed, higher educational levels, the loss of a child or a spouse and the cause of death also had a significant effect on the severity of PG symptoms. The PG-13 score of people whose loss was the result of traumatic causes was significantly higher than those whose loss was due to medical reasons. Gender and place of residence did not have a significant effect on PG severity and subjective family economic status was unrelated to the level of PG symptoms (He et al., 2014). Contrary to the Chinese study, a Danish nationwide prospective cohort study (Nielsen et al., 2017) found that complicated grief symptoms were predicted by severe pre-loss grief symptoms, being a partner and low educational level, but not predicted by age and gender.

### Stigmatized Deaths

People bereaved by DRDs often have feelings of shame and guilt for not having been able to help, as well as for why the drug problem started (Dyregrov & Selseng, 2021; Titlestad et al., 2019; Titlestad, Mellingen, et al., 2020). Previous research has shown that different ways of dying have different social status and that deaths by suicide and drugs are examples of low-status deaths. The stigma surrounding this mode of death may therefore complicate the bereavement processes (Wilson et al., 2017). If grief is not acknowledged in society because it is stigmatized (Doka & Martin, 2002), a high level of stress and feelings of shame, combined with social isolation, may increase the risk of PGD (Li et al., 2018).

The social distancing and discriminatory behaviors from those who look down on the stigmatized group (Henderson & Gronholm, 2018) often result in self-stigma, because the individual who experiences being discredited by others internalizes the social stigma (Corrigan & Rao, 2012). Proper support from informal networks (families, friends, work-colleagues, etc.) may counteract negative consequences of loss and be of great importance for coping with grief (Lobb et al., 2010). However, previous research has shown that there are several challenges, both for providers and receivers of social support, especially after unnatural deaths (Dyregrov & Dyregrov, 2008; Lakey & Orehek, 2011; Nurullah, 2012).

### A Need for Research

Due to the vast numbers of people being bereaved by DRDs and the potential for serious consequences, the field is in urgent need of knowledge about how bereaved people cope with grief. In this paper, we will examine how DRD bereavement relates to the risk of developing PGD and if the predictors and associations resemble previous studies of unnatural deaths. A key question is if and how often the grieving process becomes complicated (prolonged). As there is very little knowledge of the complexity of these bereavement processes, we will:

1. Estimate the prevalence of PG symptoms in a total sample of DRD-bereaved family members as measured by PG-13
2. Investigate which predictors are key to explaining high scores on PG-13 by performing a multivariate linear regression
3. Examine PG-13 sum scores across different sub-samples related to time since death by conducting a one-way between-groups ANOVA

## Method

### Design

This study adopted a cross-sectional, descriptive, correlational design based on data from a nationwide Norwegian study obtained from the ”Drug death related bereavement and recovery project“ (called ”The END-project’).

### Setting

The END-project was launched in the spring of 2017 at the Western Norway University of Applied Sciences. Its primary objective was to contribute to a greater understanding of the consequences and the care needs of people bereaved by DRDs and, if needed, to improve measures of help and support.

### Procedure

From March 2018 until the end of December 2018, we invited family members and close friends bereaved by DRDs to participate in the project. A flyer detailing the project was sent to all Norwegian municipalities’ public email addresses. We also contacted governmental and non-governmental personnel associated with organizations working with those affected by drug use. We disseminated information about the project through municipal medical officers and crisis responders nationwide, using research networks and professionals in clinical practice, participation in conferences, various media channels such as television, radio and social media (Facebook and Twitter), as well as via snowball recruitment methods. Participants were invited to complete a questionnaire, either on paper via post or digitally via email. The participants received an email reminder after 14 days.

### Participants

The inclusion criteria claimed that the participant was 18 years old or older, had lost a family member to a DRD at least three months before recruitment, and spoke Norwegian.

A total sample of 240 family members was enrolled. We excluded five participants who had more than 25% of missing responses in relation to the PG-13 questionnaire. In addition, one participant was excluded after normality analysis because of scores far above the other participants. The demographic characteristics of the participants are summarized in Table 1. The 234 participants were ethnic Norwegians and included family members who had lost either a female (*n* = 58, 24.8%) or a male (*n* = 176, 75.2%) relative. The average age of the deceased was 31.3 (M = 30, SD = 10, range 15–68). The income level in Norway is high, as was the income of the respondents. The average annual household gross income for Norwegians in 2018 was within the range mean score of $61,000–91,000 (26% of the participants) for annual household gross income.

**Table 1.** Overview of Participant Characteristics (*n* = 234).

Variables	*n*	Mother	*n*	Father	*n*	Sister	*n*	Brother	*n*	Daughter	*n*	Son	*n*	Other family member^a^
Relation, %	76	32.5	17	7.3	69	29.5	9	3.8	22	9.4	2	.9	39	16.7
Age, mean	73	59.2	17	60	68	39	8	41.5	22	33.1	2	33.5	37	46.4
Level of education, %	76	100	17	100	69	100	9	100	22	100	2	100	38	97.4
Primary school	7	9.2	3	17.6	4	5.8	2	22.2	4	18.2	0	0	7	17.9
High school	32	42.1	4	23.5	18	26.1	5	5.6	8	36.4	1	50	18	46.2
College/university	37	48.7	10	58.8	47	68.1	2	22.2	10	45.5	1	50	13	33.3
Closeness with the deceased, %	75	98.7	16	94.1	69	100	9	100	22	100	2	100	38	97.4
Very close	73	96	14	82.4	62	89.9	6	66.7	14	63.7	1	50	33	84.6
Close	1	1.3	2	11.8	4	5.8	1	11.1	4	18.2	1	50	5	12.8
Somewhat close	1	1.3	0	0	3	4.3	2	22.2	4	18.2	0	0	0	0
Months since loss, mean	75	83.5	17	61.9	69	105.6	9	158.8	22	93.9	2	120	39	111.9
Years of use of narcotics, mean	75	10.6	15	7.5	67	14.6	9	11.1	22	24.2	2	24	35	13.8
Sick leave after the loss, %	75	75	17	70.6	69	63.8	9	44.4	22	63.6	2	0	38	17.9

^a^Grandparents, spouses and other family relations.

### Measurements

The END-project survey consisted of 22 background variables and 79 variables from different questionnaires (standardized and open questions). Our theoretical basis and research questions formed the backdrop for our choice of variables.

#### The Background Variables

included those described as predictors in previous studies: gender, level of education, annual household gross income, time since loss, relational closeness, years of drug use before death and sick leave after death. Variables like relational closeness were rated on a five-point Likert scale, scoring 1–5 (e.g., ‘Not at all close’ to ‘Very close’).

#### Prolonged Grief Disorder**-**13

(PG-13) (Prigerson & Maciejewski, n. d.) is a criterion-based diagnostic tool developed by Prigerson et al. (2009) that assesses the symptoms of PG. The instrument consists of 13 items and is scored as a continuous measure by summing the 11 symptom items (cognitive, behavioral and emotional), i.e., the dichotomous time and functional criteria are not included in the sum-score but must be fulfilled for the PGD diagnosis. The items are rated on a five-point Likert scale, scoring 1–5. Four items range from ‘Not at all’ to ‘Several times a day’, while the next seven items are rated on an intensity scale ranging from ‘Not at all’ to ‘Overwhelmingly’ (Prigerson & Maciejewski, n. d.). The total score ranges from 11 to 55, with higher scores indicating more severe grief symptoms. There is no official cut-off score, but a Swedish research group including Holly G. Prigerson suggested a preliminary cut-off score of 35 or more to meet the diagnostic criteria for PGD (Pohlkamp et al., 2018). The psychometric properties of the PG-13 has been tested in several contexts e.g. Sweden, another Nordic country. Pohlkamp et al. (2018) showed that the PG-13 was a reliable scale for the assessment of individuals at risk for developing PGD with high scores on internal consistency. In addition, “the associations between the PG-13 total score, the level of self-reported symptoms of depression, anxiety and PTSD provided evidence in support of the instrument’s concurrent validity” (Pohlkamp et al., 2018). Internal consistency for PG-13 in the current study was good; Cronbach’s alpha (α) .9.

#### Special Grief Questions

(Dyregrov et al., 2019) examine various experiences after losing a next of kin to a DRD. SGQ is not a scale with a sum score or a cut-off score. Instead, the 16 single items measure aspects of anxiety/fear of death, anticipated grief, self-condemnation/stigma/guilt/shame, ambivalence (relief/guilt for feeling relieved) and disenfranchised grief. The present study included items that examine fear of death, stigma, complex emotions such as guilt, relief, blame and shame, and open and closed communication items. The items are rated on a five-point Likert scale ranging from ‘Almost never’ to ‘Almost always', scoring 1–5, with higher scores indicating high levels of grief symptoms.

#### The General Health Questionnaire-28 (GHQ-28)

(Goldberg, 1978) is a self-administered, 28-item screening tool used to detect those likely to have or be at risk of developing psychiatric disorders. One item was included: ‘Been thinking of the possibility that you may do away with yourself?’. The item is rated with a four-point scale indicating the following frequencies of experience: ‘Not at all’, ‘No more than usual’, ‘Rather more than usual’ and ‘Much more than usual'.

#### The Assistance Questionnaire

(Dyregrov, 2002) has previously been used to study people bereaved by unnatural deaths. This instrument contains 22 questions assessing how bereaved people report their experiences and their need for help and support. The AQ is not treated as a scale with a sum score or a cut-off score. We analyzed two items: ‘I have experienced that others have withdrawn’ and ‘I have withdrawn more from others’. The items are rated on a five-point Likert scale ranging from ‘Not at all’ to ‘To a great extent’.

### Statistical Analysis

Statistical analyses were performed using IBM SPSS Statistics version 26 (IBM Corp, 2019).

Continuous variables were described by means (M), medians (Md), standard deviations (SD) and range, whereas frequencies and percentages described categorical variables. The PG-13 sum-score was chosen as the dependent variable since the prevalence of PG symptoms and predictors for high levels of symptoms constitute the primary outcome of our analyses. At least 75% of the PG-13 items had to be completed in order for participants to be included in the study. For participants with less than 25%, missing scores were imputed by replacing them with the individuals' mean for all items completed. We replaced one missing value for eight participants, two missing values for one participant and three missing values for one participant. No replacement was provided for background variables, SGQ or AQ items. A multicollinearity check of SGQ and PG-13 items identified no collinearity issues as Variance Inflation Factor (VIF) values were below three.

We conducted descriptive analyses of the demographic characteristics and mean sum scores for the PG-13 scale. Pearson’s (*r*) was created to screen for associations between PG-13 and the potential explanatory variables. We performed a multivariate linear regression including statistically significant variables at a .05 significance level in the correlation analyses. Unstandardized regression coefficients (B) with 95% confidence intervals (CI) and standardized regression coefficients (*β*) with corresponding *p*-values are presented for the regression analyses. A one-way between-groups ANOVA was conducted to examine the impact of time since death on PG levels, as measured by PG-13. The scores on time since death were divided into nine groups (Group 1: 3–12 months; Group 2: 13–24; Group 3: 25–48; Group 4: 49–72; Group 5: 73–96; Group 6: 97–132; Group 7: 133–168; Group 8: 169–216; Group 9: 217 and above). Eta squared was calculated to determine the effect size for the results of the post hoc tests in the ANOVA.

## Results

The total sample average PG-13 sum score was 28.4 (SD = 9.5, range 11–55). Table 2 shows that the mean sum scores were 30.7 (SD = 8.9, range 15–49) for parents, 26.9 (SD = 9.4, range 14–54) for siblings and 27.3 (SD = 9.2, range 11–47) for children. The mean score was higher for female participants than for male participants. 60 participants (26%) had scores equal to 35 or higher (31.2% of the parents, 21.8% of the siblings and 20.9% of the children).

**Table 2.** Prevalence of PGS as Measured by PG-13.

Relation	*n*	Mean	Md	SD	Range
Parent	93	30.7	31	8.9	15–49
Mother	76	31.1	31	8.3	15–48
Father	17	29.1	28	11.2	15–49
Sibling	78	26.9	25.5	9.4	14–54
Sister	69	28	27	9.3	14–54
Brother	9	18.2	17	4.7	14–29
Child	24	27.3	27	9.2	11–47
Daughter	22	27.9	28.5	9.2	11–47
Son	2	21	21	8.5	15–27
Other family member^a^	39	26.8	24	10.4	11–55

^a^Grandparents, spouses and other family relations

Six of the seven included SGQ items correlated with PG-13. The item ‘Others blame me for the death’, which intends to measure stigma, did not correlate. Other variables that measure stigma, such as ‘I blame myself for the death’ and ‘I feel that others think that I have nothing to mourn’ correlated positively with the PG-13 sum score. Of the SGQ items, only ‘Feeling relieved for the death’ correlated negatively with high levels of PG symptoms, indicating that low levels of relief are associated with high levels of PG symptoms, though ‘Feeling guilt for feeling relieved’ correlated positively. Associations (and *p* levels) between symptoms of PG and selected contributing variables are shown in Table 3. Correlation analyses were used to screen for associations and 15 variables were included in the standard linear regression analyses. The R Square for the model was .545, denoting that the model explains 55% (statistically significant *p* <.001, F = 18.904) of the total variance of the dependent variable sum PG-13. The strongest associations were found between a high score on PG-13 and the variables’ months since the loss’, ‘suicidal thoughts’ and ‘withdrawal from others’.

**Table 3.** Predictors of High Scores on PG-13 as Estimated by Correlations and a Standard Linear Regression Model.

		Correlations		Standard Regression			
Variable	*n*	*r*	*p*	*β*	B	95% CI	*p*
Gender	234	−.17	**.008**	−.07	−1.79	(−4.21, .63)	.146
Level of education	233	−.14	**.037**	−.08	−1.14	(−2.50, .22)	.101
Annual household gross income	230	−.19	**.004**	−.06	−.40	(−1.08, .29)	.252
Months since loss	233	−.44	**<.001**	−.36	−.04	(−.05, −.03)	**<.001**
The closeness of kinship with the deceased	231	−.16	**.016**	−.09	−1.01	(−2.07, .04)	.060
Years of drug use before death	225	−.09	.185				
Sick leave after death	232	.24	**<.001**	.07	1.25	(−.62, 3.11)	.188
Fear disturbed night sleep^a^	231	.34	**<.001**	.11	.69	(.04, 1.35)	**.039**
Relieved^a^	229	−.14	**.039**	−.11	−.73	(−1.47, .02)	.057
Guilt for feeling relieved^a^	229	.19	**.004**	.13	.84	(.13, 1.55)	**.022**
Others blame me^a^	230	.05	.459				
Blame myself^a^	232	.42	**<.001**	.12	.81	(.12, 1.50)	**.022**
Ashamed to talk openly^a^	232	.14	**.030**	.08	.55	(−.14, 1.25)	.117
Nothing to mourn^a^	230	.27	**<.001**	.03	.01	(−.67, .73)	.929
Suicidal thoughts^b^	233	.43	**<.001**	.32	4.10	(2.84, 5.37)	**<.001**
Others have withdrawn^c^	231	.26	**<.001**	.09	.80	(−.11, 1.73)	.086
I Have withdrawn^c^	230	.33	**<.001**	.14	1.17	(.34, 2.00)	**.006**

β = standardized estimated regression coefficient; *B* = estimated regression coefficient

^a^Single item from SGQ.

^b^Single item from GHQ28.

^c^Single item from AQ.

Bold values indicate results at a .05 level

**Figure 1.**
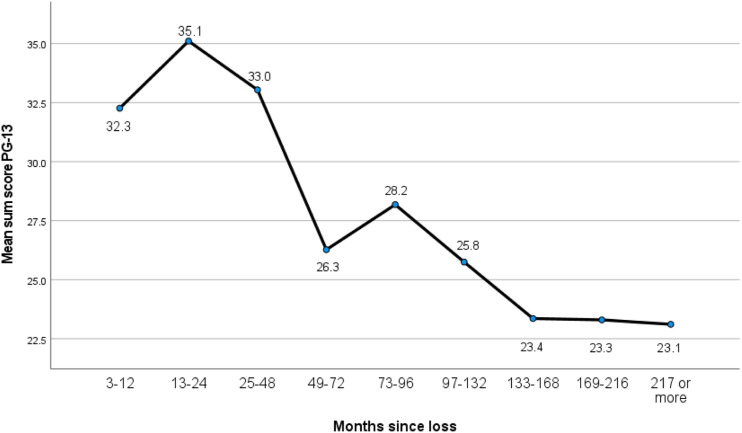
One-way between-groups ANOVA with post hoc tests.

There was a statistically significant difference at the *p* < .05 level in PG-13 mean sum scores for the nine’ time since death’ groups: *F* (8, 224) = 8.5, *p* <.001 (Table 4). The actual difference in mean scores between groups was substantial, following Cohen’s classification (see Pallant, 2013, p. 264). The effect size, calculated using eta squared, was .23. Figure 1 shows that the highest level of PG symptoms was among the participants in the group who lost a family member one to 2 years ago. Levels of symptoms decreased in line with time since loss, except for the finding that participants who had lost a family member six to 8 years ago had higher PG symptom levels than the group that were bereaved four to 6 years ago.

**Table 4.** One-Way Between-Groups ANOVA With Post Hoc Tests.

	Sum of squares	Df	Mean square	F	*p*
Between groups	4897.181	8	612.148	8.547	<.001
Within groups	16,043.823	224	71.624		
Total	20,941.004	232

## Discussion

We have studied the prevalence and predictors of PG symptoms in an unstudied group of mourners after unnatural deaths – people bereaved by DRDs. An important context to the results is that the average age of the deceased in our sample was only 30 years and the mode of death was unnatural, i.e., sudden, unexpected, violent, self-inflicted and often premature. This discussion will focus on the prevalence of PG and its connection to DRD, triggering a ‘special grief’. Social isolation and its links to suicidal ideation will also be discussed in relation to the risk of PG. Based on our results, the discussion questions whether ‘time heals all wounds’.

### High Prevalence of PG Symptoms

In line with previous SRs and meta-analyses (Djelantik et al., 2020; Kristensen et al., 2012; Lobb et al., 2010), our results documented a high prevalence of PG symptoms compared to grief after natural deaths (Christiansen et al., 2020; Lundorff et al., 2017; Rosner et al., 2021). The prevalence above tentative cut-off scores for PGD was 26% in our study, compared with studies of unnatural deaths showing prevalences of 40% (Kristensen et al., 2010), 50% (Djelantik et al., 2020) and 70% (Dyregrov et al., 2015). However, in our study, PG-13 was used, whereas other studies applied the Inventory of Complicated Grief which has less stringent criteria for PG symptomatology. In the following, we will discuss and try to explain why certain demographic variables, strains before and after the death and time since death are associated with PG after DRDs.

First, we found that higher PG symptoms scores were prevalent among females, parents (both genders), siblings and children of the deceased. Whereas some studies have documented more grief symptomatology among bereaved women than men (Hardison et al., 2005), others have not (He et al., 2014; Nielsen et al., 2017). One explanation for the higher grief scores found among women may be related to traditional gender-based roles of responsibility and caring, linked to the findings that families engage for years pre-death to try to save children struggling with drug problems (Christiansen et al., 2020). Titlestad, Mellingen, et al. (2020) highlighted this phenomenon as extended parenthood. Also, such intense and close follow-up pre-death may be connected to previous findings that close, supportive, confiding, and dependent relationships with the deceased are associated with an increased risk of complicated grief (Bonanno et al., 2002; Carr et al., 2001; Prigerson et al., 2000). In addition to increased responsibility and caregiving pre-death, women’s tendency to ruminate more than men (Johnson & Whisman, 2013) may also be relevant. Finally, the increased prevalence of PG symptoms in siblings and children may be related to their younger age, bringing greater difficulties in understanding such losses and in processing their own and others’ strong emotions (Doka, 2000; McCarthy & Jessop, 2005). Struggling with PG has also been shown to affect family relationships, therefore PG reactions in mothers may increase the risk of PG in bereaved children (Morris et al., 2016).

### The ‘Special Grief’ Increases the Risk of PG

The grief of people bereaved after DRDs has previously been termed the ‘special grief’, referring to a theoretical model of the complex interplay of possible factors (drug use, anticipatory grief, stigma, ambivalent feelings like shame, guilt and relief, and disenfranchised grief) influencing the bereavement processes (Dyregrov et al., 2019; Titlestad, Mellingen, et al., 2020). We found many strong associations between PG symptoms and contextual and personal relations and interpersonal processes. First, high grief scores were associated with a strong, sleep-disturbing, pre-loss fear of death (*anticipated grief*). Self-blame, shame and guilt, especially the ambivalence of guilt for feeling relief, stood out after death. Previous research has documented that feelings of guilt, shame and self-blame are predictive of more complicated grief processes (Dellmann, 2018; Li et al., 2018). Such feelings are often connected to having failed to protect the deceased or related to things one has said or not said, done or not done, to the deceased. An important finding in our study is that bereaved family members who only experienced relief after the death had fewer grief symptoms than those who experienced relief and guilt for feeling relieved. The ambivalence (Elison & McGonigle, 2003) may make it difficult to oscillate between loss orientation and continuing with life in a healthy way (Stroebe & Schut, 1999). Thus, the processing of grief will be disturbed, causing PG symptoms. In general, feelings of relief after a close person’s death are not accepted in society and are taboo to say out loud. Bereaved people who experience such feelings may therefore be left alone in *ambivalent rumination* that is documented to increase and prolong grief reactions (Eisma et al., 2013). Those who solely felt relieved by the death and experienced fewer PG problems may have detached themselves from the deceased before the death and may have had social networks supporting them to believe that death was the only and best outcome (Dyregrov & Selseng, 2021).

Another finding was that bereaved people who experienced that *others belittled their loss* had an increased risk of PG. This finding may reflect what Doka (2002) termed *disenfranchised grief*, i.e., when grief that is not acknowledged, either by one’s network or by society in general, does not ‘deserve to be grieved over’. Disenfranchised grief can also be self-imposed and linked to the self-blame we identified among participants, through those bereaved by DRDs taking on the stigmatizing social and cultural norms and attitudes of those around them. Indication of stigmatizing attitudes of people in social networks may be linked to the association between experiencing that *other people withdrew* and increased the likelihood of higher scores of PG symptoms. Corrigan and Rao (2012) drew attention to the concept of ‘public stigma’, which refers to the common stereotypes in the general population. In our context, this means that people bereaved by DRDs themselves are stigmatized, as has been documented elsewhere (Dyregrov & Selseng, 2021). The family is ‘contaminated’, associated with illegal and criminal activity via their relative’s drug use (Corrigan & Rao, 2012).

### Social Isolation Increases the Risk of PG Symptoms

The strongest associations were found between high levels of PG symptoms and months since the loss, withdrawal from others and suicidal thoughts. This is in line with Li et al. (2018), who stated that when grief is not recognized because it is stigmatized and when there is a high level of general stress and feelings of shame combined with social isolation in survivors, it will increase the risk of developing PGD. The strong association between high PG symptom scores and withdrawal from other people is a telling finding that may be both a cause and an effect of the difficulties experienced by bereaved people. A bereaved person will often withdraw from family and friends who utter negative and outrageous comments (Dyregrov & Selseng, 2021). Thus, a lack of social support may increase the possibility of isolation among those bereaved by DRDs and, as such, may increase the possibility of more health and social challenges. In a nationwide study of those bereaved by suicides, accidents and Sudden Infant Death Syndrome, Dyregrov et al. (2003) found that self-isolation was the main predictor of complicated grief reactions, general psychological health and trauma reactions. Whereas Dyregrov introduced the concept of ‘social isolation’, Smith et al. (2020) used ‘social disconnection’ to describe the social withdrawal of others. The concepts imply that bereaved people withdraw from social contact either because they have experienced negative comments, because they feel they reduce the joy of social gatherings, or because they are in fear of being overwhelmed by grief and have to spend a lot of energy masking their emotions. Both research groups demonstrated that this phenomenon was closely associated with and was a factor that maintained PG (Dyregrov & Dyregrov, 2008; Dyregrov et al., 2003; Smith et al., 2020), and may well also be connected to the following finding of suicidal ideation.

### Suicidal Ideation to be Taken Seriously

The strong association between PG symptoms and suicidal ideation among people bereaved by DRDs in our study indicates that the high levels of PG symptoms must be taken seriously. Previous research into unnatural deaths has shown that complicated grief is linked to suicidal thoughts and a recent SR revealed that people bereaved by suicide, another stigmatized group, had the highest incidence of suicidal thoughts compared to other bereaved groups (Molina et al., 2019). Stigma, isolation tendency and avoidance behavior were found to be among the risk factors. Other studies have also shown that bereaved people have an increased risk of suicide, especially in the first year after a loss (Guldin et al., 2017). The link between suicidal thoughts and PG was also documented in a study of 149 bereaved people who suffered from PG. The researchers found that two-thirds reported suicidal thoughts and 9% had attempted suicide (Szanto et al., 1997). The strong correlation between high levels of PG scores, serious suicidal thoughts and lack of meaning in the lives of bereaved people is also documented by other grief researchers (Prigerson et al., 2009; Shear et al., 2011). Furthermore, this may be linked to rumination about failure to save their loved ones, self-isolation and stigmatization from social networks (Dyregrov & Selseng, 2021). Thus, there is a danger that PG and suicidal thoughts are mutually reinforcing and must both be given preventive attention, i.e., increase meaning making among bereaved people to reduce the incidence of PG symptoms (Neimeyer et al., 2006).

### Does Time Heal all Wounds?

The phrase’ time heals all wounds’ may be first attributed to the Greek poet Menander (300 B.C.) who stated that ‘time is the healer of all necessary evils.’ This has also been people’s mantra when approaching grieving people. In keeping with this ancient truth, our study documented that the highest level of PG symptoms was found among participants in the group who lost a family member one to two years ago. Findings generally showed that grief symptoms decreased with time since loss, although the group of participants who had lost a family member six to eight years ago had higher levels of PG symptoms than the group who were bereaved four to six years ago.

Support over time is of great importance in grieving. However, much grief research documents that there is a large discrepancy between bereaved people’s need for support over time and network members and professionals’ understanding of this need (Aoun et al., 2019; Bartik et al., 2015; Dyregrov, 2003; Turnbull & Standing, 2016). Unfortunately, bereaved people often experience a time paradox connected to support and help. This implies that networks and helpers give the most attention and offers help just after death, when bereaved people are in a state of shock and are unable to take advantage of the various kinds of help. As time goes on and the shock subsides, the vulnerability of bereaved people increases but they also experience that friends, family and professional support is withdrawn in the belief that enough time has passed (Dyregrov & Dyregrov, 2008). The reality is often that bereaved people then feel alone and have a great need for support. The paradox of helping attitudes may explain the highest prevalence of PG symptoms between the first- and second-years post-loss.

In addition to the finding that high PG scores are strongly associated with time since death, the figures also show that many bereaved people experience serious grief symptoms that last much longer than expected by both their own support network and society in general (Dyregrov & Dyregrov, 2008). After DRDs, bereaved people need a long time to process and ‘heal’ the ‘special grief’, especially personal feelings of failure, and to feel reintegrated into society. In line with findings from other research on grief and crisis (Dyregrov et al., 2015), we found that higher income and education levels were associated with lower levels of PG symptoms. This might be explained either as a cause or an effect, or be combined (Burrell et al., 2018, 2020). Low household income and less education may be linked to less ability to elicit adequate help pre- and post-loss, and multiple concurrent health challenges combined with a more turbulent home environment. Post-loss, there may also be potential reasons for reduced probability of educational attainment in bereaved children, connected to impaired concentration, lower self-efficacy and lower school attendance (Burrell et al., 2020; Dyregrov & Dyregrov, 2008).

### Implications of Findings

The findings show that the consequences of DRDs are serious and long-lasting for many bereaved family members. This knowledge must be communicated clearly to reduce the chances of marginalization of a large group of grieving people.

Helpers in health and welfare services should be educated about the ‘special grief’ after DRDs and the risk of, and the deliberating consequences of, PG to minimize the prevalence of PGD. In particular, professional grief therapists should be supported to increase their competencies to be able to diagnose PGD (Prigerson et al., 2021) and to treat those bereaved who fulfil the criteria for PGD (Shear et al., 2019). The PGD diagnosis in the ICD-11 will hopefully contribute to professional interest in the field and the provision of need-related help to bereaved people beyond the first-year post-loss.

The social networks of family, friends, work colleagues and neighbors also need to be educated about the hurtful consequences of stigmatizing communication and to overcome their uncertainties in relation to supportive communication and support over time (Dyregrov & Dyregrov, 2008; Dyregrov & Selseng, 2021; Titlestad, Stroebe, & Dyregrov, 2020). It is also essential to make people aware that stigma exists; increased knowledge as to why it occurs and how it is transmitted in society can help remove stigma of people bereaved by DRDs.

Finally, people bereaved by DRDs will gain from learning that their ‘special grief’ reactions are common and normal close to their loss, but that they can and should be helped by competent professionals if their grief does not improve.

## Methodological Issues

Cross-sectional research has several limitations (Johnson & Christensen, 2016, p. 401). Although it is advantageous that data relating to many people can be collected within a short timeframe, our data relate to an extensive time period, in order to extend the sample size of a population that is difficult to recruit. This may result in different contextual conditions for the bereaved participants, as well as making it challenging to determine whether the exposure or the outcome comes first. Therefore, when using the concept predictors resulting from regression analysis, associations rather than causality are highlighted. Although serious life events are often remembered with great clarity, recall bias might have occurred, as many participants lost their loved ones a long time ago and the average time since loss was considerable. However, items included in the analysis essentially relate to the present, which may have strengthened the validity of the results. We argue that through our knowledge of bereavement and unnatural deaths, we chose variables that were important to examine in relation to the population studied. Still, since we were researching an unstudied field, it was challenging to identify the most critical predictors for investigation. The outcome measure PG-13 is a standardized questionnaire that is used by clinicians to diagnose prolonged grief disorder in bereaved people. We have therefore not made assumptions about a disorder, but rather, have described levels and prevalence of prolonged grief symptoms referring to sum-scores from the instrument.

Our study is not a clinical or experimental study. The aim of the study is descriptive rather than to test a hypothesis. Moreover, it is based on a convenience sample and the total population of bereaved after DRD is unknown. Thus, a power analysis is not relevant, possible or correct for this study. A convenience sample, depending on the recruitment method, limits the findings' generalizability. Despite these limitations, this project involves what is to date the largest sample of family members bereaved by DRDs, which increases the relevance of the findings. We argue that the results must be appraised in light of their contextual information, namely, the cultural context and the population included, the phenomena of our research interest and how participants were recruited.

## Conclusion

This study shows that many family members suffer from high levels of PG symptoms after DRDs. This implies that time does not always ‘heal all wounds’, healing takes longer than most people assume and a faster healing process is connected to several factors. People bereaved by DRDs need attention from professionals, social networks and peers to mitigate the development of PGD and to enhance meaning-making and quality of life. On a societal level, knowledge must be distributed about the extent to which bereaved people may have suicidal thoughts, experience self-stigmatization and isolation connected to ‘unworthy’ deaths and the need to reduce stigmatization of people who use narcotics.
